# Between commerce and care: intimate personalization, authenticity, and the ambivalences of outsourcing funeral work

**DOI:** 10.3389/fsoc.2026.1690172

**Published:** 2026-02-18

**Authors:** Roberta Sassatelli, Rossella Ghigi, Federico Boni, Elia A. G. Arfini

**Affiliations:** 1 Department of History and Cultures, Università degli Studi di Bologna, Bologna, Italy; 2 Department of Education Studies, Università degli Studi di Bologna, Bologna, Italy; 3 Department of Social and Political Sciences, Università degli Studi di Milano, Milan, Italy

**Keywords:** authenticity, commercialization, consumption rituals, emotions, funerals, intimate personalization, Italy

## Abstract

The paper considers the way commercialization is negotiated in important consumption rituals such as funerals by mourners. Focusing on Italy, we start by providing data about funeral participation, and propose that there is a tension between affective and commercial personalization which informs all stages of the social management of death and the encounter between the mourners and the funeral industry. We look at how the organization and purchase of funeral services is managed by mourners, explore the wake and the way the dead body is handled between families and industry, and finally concentrate on the funeral ceremony with particular attention to how emotions are marshaled, and personalization introduced. We conclude by discussing the ways in which funerals may be considered by mourners as successful and argue that positive experiences rests on valuations of authenticity associated with affective personalization and therefore reveals the ambivalence of the commercialization of funeral rituals.

## 1 Introduction

Although highly differentiated from one culture to another, funeral rites are cultural universals that help us to greet the dead, to let them go beyond life by sanctioning their passing, and to facilitate mutual recognition among the living affected by their death (Kearl, 1989). Grief has been individualized in the Global West, and often the discourse on death addressed to the bereaved emphasizes that “there are no rules in grief, there are no predetermined stages or paths, and that grief is entirely personal to the individual” (Walter and Bailey, 2020, p. 178). Mourning is a practice “to mutually acknowledge an intimate experience” (Guillard, 2017, p. 481). Yet, recent critiques (McCarthy et al., 2023) argue that grief, rather than being a solitary, psychologised experience, is better understood as relationally embedded within family and community networks, shaped by moral obligations, shared memories, and ongoing collective practices. In this context, the funeral remains a public moment of expression of grief which offers a shared and highly ceremonial occasion for mutual recognition of feelings. As a public ritual, the funeral is perceived as a momentous accomplishment of private emotions, even though today funerals are organized by commercial organizations that are offering an all-encompassing commodified service. The commercialization of key passage rituals which were once more community-based, intimate and/or traditional is accompanied with the staging of deep feelings that are presented as personal and authentic. Such rituals, now fashioned as consumption rituals and largely integrated in consumer culture dynamics of normalization (Sassatelli, 2007) are an important territory where commercialization and emotions momentously meet.

Emotions, feeling, affects have become an important object of study within sociology, revealing the social nature of human emotions, and the emotional nature of social phenomena (Ahmed, 2004; Bericat, 2016; von Scheve, 2018). All in all, there is a growing recognition that “we feel in socially arranged ways (more) than we think we do” (Hochschild, 2003, p. 76). Feeling rarely bubbles freely and spontaneity too has its conditions: it requires work, some form of reflexivity, concertation with those also present, certain happy structural conditions in the way interaction is organized. Funerals are recognized as occasions where emotions are socially governed both through the operations of an industry (which helps facilitating the funeral ritual through commodities and services), and through the active participation of the mourners (who find ways of expressing feelings which are socially shared and recognized). Thus, the commercialization of the funeral ritual participates in a general trend whereby the realization of emotional projects is increasingly carried out within the market and, more specifically, through the purchase of commodities thick in emotional value (Illouz, 2018).

There is a wealth of literature on commercialization and emotions: scholars of consumption have been increasingly interested in emotions and in the only apparently contradictory concurrence of rational and emotional practices, market and intimate life, commodities, and personal relations (Alaluf and Illouz, 2019; Carù and Cova, 2007; Chaudhuri, 2006; Hansen et al., 2007; Illouz, 2018; Sanders, 2009; Zelizer, 2005, 2009). What is largely admitted is that there is a growing interplay between intimate emotions and commercial culture. What is ordinarily felt as priceless—such as people, love, and care—increasingly needs to be priced (Zelizer, 2005), Contemporary capitalism has been defined as “emotional capitalism” precisely because the boundaries between the economy and intimate life, the public and the private are continuously renegotiated: the market penetrates intimate life and, conversely, emotions are increasingly called upon in corporate relations (Illouz, 2007). Hochschild (1979), in particular, has noticed that the more bureaucratic and commodified society is, the more inflated positive expressions of feeling, for which reason they are taken less seriously. This is part of a wider cultural trend that with the post-war decades of sustained economic growth and the “revolution of consumerism” has given way to what Taylor (1989) has called the “culture of authenticity and expressivity” in Western societies. Authenticity has been defined as a modern value and ideal that resulted from the experience of inauthenticity and alienation in Western modern, highly commercialized, society (Banet-Weiser, 2012; Vannini and Williams, 2009; Wang, 1999, 2000). While the quest for authenticity is centuries old, authenticity has increasingly become a precious commodity itself and at the same time it is one of the fundamental stakes in consumption. The quest for authentic goods and consumption experiences plays an important role in today’s marketplace and in sociological research on tourism, food, music and cultural production, and even urban environment (MacCannell, 1976; Wang, 1999; Arfini, 2019; Fine, 2004; Grazian, 2003; Koontz, 2010; Peterson, 2005; Piazzoni, 2018; Sassatelli and Arfini, 2017; Sassatelli, 2019).

In this context, captured by emotional capitalism, the funeral industry lives through a paradoxical configuration between prices and emotions. It has to offer an efficient service for mourning in ways which ultimately will help the mourners to reconcile with the loss and the pain, a standardized service that nevertheless is true to intimate memories which define the singularity of the deceased, an all-around service which still allows space for the feelings of the mourners to be expressed in ways that they may deem authentic. Thus, in this paper we consider the way the commercialization of funeral services is negotiated via various forms of emotion work (Hochschild, 2003) by mourners and their ways to participate to funeral rituals.

Data presented is part of a larger research project on Italy, death, and the funeral industry. The project explored the beliefs, attitudes, and behaviors of Italians regarding death and dying. Many aspects were investigated as to attitudes towards death as well as experiences of bereavement and mourning. In this paper, we investigate funeral rites and ceremonies foregrounding the actors involved, in particular the mourners and explore their experience with the funeral industry. The research has been conducted using several methodologies.^1^ First, a sample survey was administered using computer-assisted personal interviewing (CAPI) on a representative national sample of 2,000 cases, and bi- and multivariate analyses were accomplished. Second, 405 in-depth interviews were carried out. These narratives were collected within families conceived as survey units, so that at least two members from different generations within each family were interviewed, to maximize intra-family as well as extra-family generational and gender variety. Family units were recruited among different social groups and contexts, according to four dimensions: the territorial dimension, meaning both the region and the demographic size of cities; class, status and gender; generations; and social mobility across generations.^2^

The paper is structured as follows: in the first section, we provide data about funeral participation in Italy and lay out the basic conceptual compass that will guide our discussion: what we define as the negotiation of *outsourcing* and allowance for *insourcing*—with the ensuing tension between *commercial personalization* and *intimate personalization*. In the following paragraphs, we look at how this tension unfolds in the key moments of the social management of death: the organization and purchase of funeral services, the wake, and the funeral ceremony. Finally, in our concluding remarks we discuss ways in which funerals may be considered by mourners as successful: arguing that the positive appreciation of the quality of the funeral as a “good” one mostly rests on the value of *authenticity* associated with intimate personalization.

## 2 Funeral participation between intimate and commercial personalization

Funeral rituals in the Global West are characterized by a deep tension between the economic calculation that assigns a price to everything, to the body and its capacities, to life as well as to death, and the idea that the subject, his life, his body—whether dead or alive—are sacred, unique and priceless (Walter, 2020). To be true, embodiment and the body are increasingly conceived as plastic, but the malleability of the body is typically conceived as a subjective right, rather than itself a commodity with a price (Sassatelli, 2012; Sassatelli and Ghigi, 2024). Arguably, partly because of this, funerals are caught in a series of tensions: between commodity and emotions, private and public consumption, individual and collective mourning. In Italy too, the funeral today is caught between these dualities: on the one hand, it is outsourced by the family to commercial funeral companies; on the other, the family finds itself and recognizes itself in funerals as a network of private, emotionally-dense relationships that bear witness to the feelings for the deceased and celebrate his unique, priceless life. Ultimately, what is at stake is the authenticity of the ceremony. Authenticity has been classed into two separate types (Selwyn, 1996): authenticity as knowledge (“cool authenticity”) and as feeling (“hot authenticity”). In our research, we have dealt with hot authenticity: considering in what circumstances attending a funeral becomes a meaningful for the mourners beyond commoditization. Indeed, in a ‘good’ funeral, mourners participate to the informal performative process of creating and reinforcing a funeral’s authenticity. By focussing on such performativity, Cohen and Cohen (2012, p. 1300) show that “the process of ‘hot’ authentication is emotionally loaded, based on belief and on the commitment and self-investment on part of the participants. It is an accumulative, self-reinforcing process”.

Now, what do we mean by “authenticity” when referring to funeral consumption rituals and funerals experience? Bailey and Walter (2016) conducted an extensive qualitative data on how people experience public events through mass observation in Britain. They considered three issues raised by correspondents: accuracy, performance, and authenticity. Our use of the concept of authenticity builds on their work, but summons all three issues. It refers to the overall experience of the funeral, not just to eulogy, but also to gestures and objects. This is crucial if we are to thematize the construction of a meaningful experience as consumption, and consumption as a process which may produce authenticity as its fundamental value.

For most of the Italian population, the funeral is an important ceremony that arouses strong emotions, albeit channeled into prescribed forms (according to the Catholic tradition, as in most South-European countries). The relevance of funerals in memory and experience is supported by quantitative data. In fact, we find that 92.2% of Italians has gone to at least one funeral in their lifetime, and 80% has been to two or more funerals. Our interviews confirm strong social expectations around the involvement of the close family of the deceased in the decision-making process behind the funeral. Responsibility for organizing it generally falls on the person in the family who was already caring for dying person’s end of life, i.e., a close relative. It represents a pressing issue in family configurations, which will have to modulate the organization of this challenging task. It is, in fact, an experience faced at a time of exception and pain. Given the scarce propensity of Italian families to prepare for death—75.5% of our survey sample have never even thought about what they would or would not like at their own funeral—the responsibility of organizational choices falls on those who remain.

Certainly, funerals are highly codified ceremonies, they follow highly pre-established rituals. As such, they consist in planned processes, are likely to be sequential, tend to resist change and prescribe appropriate choices, feelings, and actions (Nations et al., 2017). As any other ritual, they need script, roles, audience and artifacts. By and large, these characteristics hold in our findings. The sequence, as appropriate for a ritual, is fairly standard. There is the moment when the body arrives, after the wake at home or in the mortuary, often accompanied by a funeral procession. There is the function, with a central role entrusted to the celebrant. There may be memories from relatives or friends. Everything is surrounded by flowers, and often accompanied by songs. Then, as the family leaves the church, there is a time of direct expression of condolence, when congregants individually express their sympathies to the close family. Finally, in many cases, the immediate family and some of the relatives and friends, go to the cemetery for burial. Especially in Sicily and Sardinia, and more generally in the South of the country, traditions persist: the wake before the funeral, still very much in use, is fundamental; the journey of the coffin from home to church is often accompanied by music or in any case greeted by passers-by; the coffin is sometimes still carried to church on the shoulders, the ceremony in church is accompanied by religious songs; and the tradition of the *Cunsolo or U cunzu*, the bringing of food to close relatives and perhaps stopping to eat with them, persists after the funeral.

As suggested, in Italy most funerals are religious ceremonies, particularly Catholic ones, and are celebrated in a church. The funeral ceremony in church is seen as a standard which is only partially modified, with the introduction of increasingly important elements of *intimate personalization* (readings, eulogies from relatives or friends, special music, special groups performing actions together, etc.). Such elements of intimate personalization in fact, as delivered through informal emotion work, may be seen as drawing on a paradigm of care which posit itself as largely alternative to commercialization (Hochschild, 2003). Indeed, they not only are generally the most appreciated aspects of a funeral, but they are also often typically understood as distinct (and sometimes even contrasted with) *commercial personalization* as related to commodities of different sorts (flowers, a particular coffin, special print images, etc.). Caswell (2011) similarly observes that even within highly ritualized religious ceremonies, mourners act as reflexive agents, selectively integrating personal elements into traditional rites to assert authenticity and relational closeness to the deceased. While we will stress more the emotional and affective aspects of such intimate personalization elements, they do reveal how personalization does not necessarily replace ritual elements but rather coexists with and subtly reframes them.

The tension between intimate personalization performed by the mourners and commercial personalization offered by the increasingly customized services of the funeral industry, and more broadly between moral and economic worth is apparent throughout. Indeed it originates and is apparent since the very first moments of the organization of a funeral, that is the purchase of funeral services.

## 3 Organizing the funeral and buying funeral services

In the purchase of funeral services, the tension between moral values—honoring the memory of the deceased—and economic values—buying a quality service at the right price—is central. In this sense, the funeral market shares some characteristics with other particular markets that have been the subject of interest within sociology of valuation (Beckert and Aspers, 2011; Beckert and Musselin, 2013) because they test the boundaries commoditization. The engagement of consumers with the funeral market is complicated by the tension between the sacredness of the individual and the practical need for externalization, which results in a commercialization of intimacy (Hochschild, 2003).

53.7% of Italians have organized the funeral of their last deceased close relative. Funerals organizers usually coincide with the dead’s close kinships, as defined by marriage, direct descent, consanguinity or affinity. Funerals are telling moments for families and friends about the relationship between the living and the dead; seen from a interrelation perspective, they are understood as an index of the quality of the relationship (Woodthorpe and Rumble, 2016). The organization of the funeral brings out a performative dimension of belonging: what emerges in most of the interviews is a confirmation that those who are expected to organize the funeral and participate from the onset are the most authentic affective bonds of the deceased. Therefore, the recognizing who has the final say over the personalization of the ceremony or on the body disposal amount to acknowledging the close and authentic bonds of belonging of the deceased.

While recognizing the importance of the personalization of the funeral, outsourcing or delegating funeral work is a necessity when the funeral organizer is confronted with a series of urgent choices, which often have to do with the management of the dead body, especially in cases when it is displayed at home. As G., a female employee living in a large northern city, recounts:

I was in a hurry for that crate with this cooler to arrive because I said ‘gosh, the heating is on’ (…) And then in the days that followed (…) I remember that every day I saw some degeneration (of the body) (…) In fact, I was terrified that the funeral would not take place by Monday. I was saying, gosh, cannot it be done already because… just think if it starts to give off some smells, right? What are we going to do? (Int. 34).

When a loved one dies and a funeral is arranged, the organizer is faced with a broad range of regulations that impose strict constraints on social expectations, mortuary technology, legal norms and, finally, financial resources.

The data collected from all those who have organized a funeral—or have personally assisted in organizing it—show that funeral services are mainly organized by funeral directors (68%) or by a priest (22%). All other possible resources (including brotherhoods,^3^ insurance companies, associations, cremation societies, the municipality) are used only residually (10%). In 75.5% of the cases the company contacted is the one that the close family has always used, who alternatively, in 24% of the cases, choose a company recommended by friends or acquaintances. The access to funeral services occurs, therefore, through the mobilization of trusted personal networks. In fact, even when the respondent had no previous knowledge of the funeral industry, in 70% of cases they followed the advice of a relative or friend. A minority (17%) simply reported that the company they chose was the closest to the place (home, hospital, hospice) where the person had died. Advertising does not seem to pay: only 0.1% said that they found the company in a newspaper, a magazine, or on the internet.

In any case, it would not be easy to advertise funeral services, i.e., a type of business that makes a profit out of the misfortune of others, relying on most common marketing tools (e.g., 3×2 offers, sponsorship [Akyel, 2013, p. 228]). It is thus preferred to rely on loyalty and personal trust networks. Presenting funeral services as an activity distant from the market profit logic is consistent with the demand: burying one’s own family member has an immeasurable value for those left behind. Overall, the judgement on the service provided by the companies was positive. Even if our informants provided reflections on the economic cost and expressed a general attitude to save money, they were mostly acutely aware of the symbolic value of their purchase: “you can say ‘spend as little as possible’, but when you are in front of the funeral home you also feel ‘what a bad person I am to say. I don’t want to spend’” (Int. 37). When purchasing a funeral service most end up justifying price preferences by expressing a form of “average” or “middling” taste (“Actually I chose something average, with some decoration, not too simple” [Int. 103]), associated with values such as sobriety, dignity, respectability. In short, aesthetic valuation is acknowledged as important, and it is associated with moral values that refer to care, orderliness, discretion, attention to detail, the professionalism of the good craftsman, and the predictability and punctuality of the service.

All in all, the commercialization of funeral services is more often than not narratively obscured by outsourcing. The first thing that is often said by family and friend who had to organize a funeral is that they had been able to delegate: “all you have to do is make a phone call and they (the funeral service company) do everything” (Int. 299). Often accounts do not go much further than this. The possibility of outsourcing the funeral work also represents the opportunity to be able to concentrate on and cope with the grief privately. The fact that “they did it all” closes the funeral work in a black box, with no possibility to enter into details about it, and precisely because of this is greatly appreciated. A further form of positive evaluation is in the appreciation of the kind of emotional work carried out by undertakers. This happens mainly through discretion—which as we will see have mainly to do with the concealment of details about the management of the corpse: “(the funeral directors) didn’t let us see grandpa because there was something bad, I don’t know what. (…) My grandmother didn’t see him either, no one did” (Int. 325). There are also cases, especially in the interviews in the South, of outsourcing the work of mourning. In such cases, the professional funeral directors take charge of details of the work of mourning traditionally carried out by friends and relatives (this is the case of the funeral directors who in Sicily bring breakfast to those who participated the wake). In these forms of externalization of intimacy (Hochschild, 2003) there are also accounts of funeral operators who provide forms of emotional support to the bereaved with gestures of closeness and attention.

On the other hand, our interviewees expressed negative evaluations towards commercial practices that are considered too competitive and marketing-oriented as well as aggressive and oriented to search for profit at the expense of people’s pain. As in Turley and O’Donohoe’s (2017) research, many interviewees noted the contrast between their own heighted emotions and the commercial interests of service providers and sometimes downplayed their expectations for empathy and emotional care accordingly.

Our interviewees’ emphasis on delegating “everything” to funeral companies mirrors a broader shift towards privatized mourning practices (Woodthorpe et al., 2022), where control over timing and participation often outweighs adherence to public ritual. As we have seen, outsourcing the organisation of the funeral to the market is a crucial aspect of families’ relationship with death, and it is also a peculiar feature of this consumer experience. When funeral services are placed on a market, when they are commercialized, that fundamental tension between the sacred (death) and the profane (consumption) is activated. As Manuela, a middle age woman from a small town in central Italy, said:

I remember the impression I had, I always said, it’s like the Postal Market (mail order) catalogue you know? That you say: I want this… I want this… in this colour. This is impression the Postal Market (mail order) catalogue made on me: an ugly thing (Int. 236).

As an exception to outsourcing the fundamental logic around the management of a funeral, subjects may want precisely to overturn—at least in part and parcel—the convenience and normality of outsourcing and insert themselves in the process of production. This allows them to more clearly claim their own direct agency, subtracting elements of the funeral from the market, and valorizing the whole ritual as a superior form of homage and exclusive closeness to the deceased. And of course, in this case the reclaiming of the mourners’ agency, as indicated in the next paragraph, occurs also through physical proximity the corpse. Outsourcing is overturned, on the ground that choices provided by the funeral industries are too commercial and consumerist, failing to take on the value of intimacy and authenticity. Broadly speaking, outsourcing may indeed look as posing a challenge to intimacy, and various elements of intimate personalization set in as a compensation to it. As we shall see, intimate personalization is fundamental in characterizing the value of the (good) funeral. An important asset of funeral directors, therefore, is not only customization, or avoiding selling a ‘one-size-fits-all’ funeral by increasing the number of options available as consumer choices. It is also flexibility and *insourcing*—or involving consumers themselves in the production of the funeral. By insourcing we mean that the funeral industry appears to have become quite capable of working with the family, relatives, and friends of the deceased in a rather flexible manner to make space for the mourners’ agency and their contribution to the services they are selling. Encouraging some form of “prosumption” (Ritzer, 2015) through insourcing is crucial. As Turley and O’Donohoe (2017) show, for professional funeral providers customizing and fine-tuning their services *de rigour*, but they are also asked to be flexible and open, and to take a step back and encourage the mourners to take care of some of the tasks involved.

## 4 The preparation and the wake of the corpse

One of the most critical—and painful—practices that follow the passing away is the preparation of the corpse. Also, in this case outsourcing is widespread:—“they did everything by themselves” is the typical comment of family and friends about the funeral operators who dealt with the dead body. This phase takes place mostly in the “backstage,” where all is professionally and routinely managed by the funeral home (in what may be defined as the “solitude of the corpse,” paraphrasing Norbert Elias, 1985). Yet, this is also the phase in which it is easier to come across an exception to outsourcing, by engaging in intimate practices as washing the dead or dressing them up, when the mourners claim a final occasion of intimacy with the deceased, that is presented by the interviewees as intensely personal and authentic.

This phase represents one of the most critical aspects because both the preparation of the dead body and the funeral wake are characterized by the central presence of the corpse. That is, matter that reminds us in such a macabre and disturbing way of the perishable nature (in fact, the very decomposition) of the body—and of the individual—(Featherstone, 1991), the limit of body projects and of identity with them (Shilling, 1993). In this case, the corpse can be seen as a disturbing presence, in the double meaning of the Freudian concept of *unheimlich*: that is, both as something familiar and unfamiliar at the same time (or as something unfamiliar that creeps into the familiar); and both as something homely that at the same time is un-homely, (the reference here, out of metaphor, is to the funeral wake set up at home: the space is that of home, but the corpse does not really belong to that space, as if the dead were not to stay at home [Royle, 2003]). This is probably the phase in which outsourcing to professionals saves the closest relatives a particularly painful and disturbing operation. After all, placing the responsibility for the body into a third party further serves to separate life from death and solidify death and the corpse (Nations et al., 2017).

If the takeover by the funeral home turns into an almost total “invisibility” of the various practices connected to the management of the corpse (“they arrive (…), they do everything by themselves, the cleaning, the dressing, the fixing” [Int. 153]), an active participation in such operations (or at least in part of them), when it is asked for, is experienced as a legitimate claim to agency which, as suggested restores a particular intimacy with the deceased. In this case, the bond with the deceased is told as stronger than the sense of discomfort which may be caused by a direct and prolonged contact with the dead body—indeed, it is sometimes experienced as a (relatively) pleasant moment.

Normally, the contribution of the relatives to the preparation of the corpse is limited to the dressing of the dead body. In the stories of many of the interviewees, this operation follows the precise will of the deceased, who had previously indicated the clothes in which he or she wished to be buried. Furthermore, this is an occupation reserved mostly to women: “usually the women of the family do it” (Int. 159). Men, if anything, deal with more “masculine” aspects, as in the case of the grandson who shaves his grandfather—in a last and exclusively male gesture of care and solidarity –: “he said to me a few days earlier “Diego, can you shave me?,” and I told him “come on, I’ll shave you later,” and then I had to do it in the hospital, when he was already dead” (Int. 389).

The dimension of the macabre is more evident in those cases when some disturbing operations managed by the “professionals” are inadvertently seen by the relatives. The disturbing experience of sight of the corpse (and contact with it) is also typical of the phase that follows the preparation of the corpse, that is, the funeral wake. The funeral wake is a common experience: more than half of the Italian population visited the place where the corpse was exposed. The chamber of rest is mostly set up in the very home of the deceased (50%) or in the hospital morgue (42%).

With the preparation and the eventual wake of the corpse, a ritual is celebrated that not only reconstructs the materiality of the body of the deceased as a node of a whole network of social relations, but also redefines through a long, silent and idle mourning time, the self of those who participate in this ritual (Foltyn, 2008; Hockey et al., 2010; Hallam and Hockey, 2001). In this ceremony the materiality of the corpse is offered to the senses of the participants, especially in relation to physical contact (touching the corpse, kissing it) and sight. Sight seems to play an essential role: it is with the vision of the corpse that the identity of the deceased is ratified and restored—or not (Synnott, 1993; White et al., 2016).

Here comes into play a rather particular aspect of the idea of authenticity, that is related to the self of the deceased (and, consequently, to that of the survivors): should we consider the corpse or the memory of the deceased while alive to be more authentic? Often the corpse is not considered to be something related to the deceased individual while alive. As in Nations et al.’s (2017) research, some people expressed the need to see the lifeless body of the beloved one as no longer central to the deceased’s identity. Indeed, in such cases the very sight of the corpse turns into an experience of estrangement: “No, it was not the same person for sure. In my opinion it was another thing” (Int. 57); “Every time I have seen people in the coffin (…) of course you recognize them because you have known them, but they are no longer the same persons” (Int. 75); “My mom (…) turned into a corpse. So, it was no longer who she used to be” (Int. 5); “He looked like another person (…) I saw on this man’s face a kind of damnation” (Int. 212).

In all these examples not only does the sight of the corpse appear as the experience of the inauthentic (“another thing,” “it was no longer who she used to be”), but it turns into a real macabre encounter: “I was impressed by rigor mortis, … that is, the rigidity of the corpse, the open mouth… it was like being in the Egyptian museum” (Int. 56); “I remember the cold skin (…). The loss of color” (Int. 46).

Alongside this uncanny experience, it is possible to find a more serene experience, where the inauthentic (“I prefer to remember him alive”) leaves room to quasi-authentic (“he seemed to be sleeping”), that is, to the recognition of the corpse as the individual while alive. In this case, the contact with the corpse is decidedly less problematic: “Many people, here, kiss their hand, their forehead, or embrace them (the dead). It is our custom here (…). I do not feel impressed by this. It is as if they were still alive” (Int. 360). According to Nations et al. (2017, p. 410) this is a defence mechanism, and the inability to distinguish between identity and body, which could hinder the pursuing of self-preservation.

The ritual of the funeral wake represents another moment when issues of authenticity are at stake.

Many of the interviewees remember the moment of the funeral wake at home as an occasion of great help and consolation, especially in relation to the closeness shown by other participants. In many cases, the vivid memory of the open doors of the house and the presence of visitors is accounted for as a (relatively) pleasant experience. The space of the house can be considered here as a “middle region” between the public and the private places, where the participants can stage their deep feelings in an intimate yet semi-public way.

Of course, the funeral wake can also be a rite that is experienced with reluctance, or decidedly refused. Many of the interviewees qualified the funeral wake as an inauthentic “staging” of the emotions, the “apotheosis of hypocrisy,” played by “actors” (Int. 77), where “everyone always asks the same questions: “What happened? And what about the children now?” (Int. 295). In the sociology of death, mourners can be categorized as primary or secondary according to how much they are affected emotionally by the death which occurred and to social expectations around it (i.e., Walter, 2020). After all, funerals are events that provide people with the chance to publicly acknowledge their relationships with the deceased and balance the appropriate expression of emotion as part of this understanding.

If the funeral wake, with its rituals and its ceremonies, can be considered as a particularly weird phase, perhaps the most painful and sorrowful moment is that of the closing of the coffin. Through our survey, we know most people prefer not to be present at the closing of the coffin—only 25% of the Italian population undergoes this extreme farewell ritual. The stories of many of the interviewees show a particularly shocking experience: “That was the most difficult moment because I realized that I would never see him again” (Int. 26); “the smell. I remember this awful smell. The coffin has to be sealed and I think they use zinc, which makes a particular smell” (Int. 20).

Since it is the last chance for contact with the dead body, the closing the coffin is the moment when people have the last opportunity to leave something with the deceased beloved. Guillard (2017) shows in her research that the managing of the deceased’s belongings is a fundamental part of grief work and starts from what relationship the departed had with some of his/her objects. This is particularly true at the onset (when a funeral is organized): the deceased’s possessions become in fact sacred as long as they crystallize his/her identity and through the reclaiming of memories are a testimony of networks of relations. In general, people in our research tend to leave with the body of the deceased objects that are recognized as being particularly significant for the deceased, such as “a pack of cards, because he always played with my grandchildren” (Int. 301), or “a prayer book, a rose and … a hat (…), something that represented her so much” (Int. 302), or even a “copy of the novel, Gone with the Wind” (Int. 240). As in Drenten et al.’s (2017) research these grave gifts appear to w restorative practices and serve as performances to reinstate what was taken to them. But our findings also show that during mourning rituals they serve as key moments to personalize some otherwise standardized and commercialized practices and perform in very tangible and memory-rich ways authenticity.

## 5 Grief management and the personalization of the funeral

Purchase and outsourcing are explicitly reframed in funeral rituals though the management of emotions and the personalization of the ceremony. By providing an occasion for public condolences and offering a last image of the deceased, funerals allow in highly ceremonial forms a space for constructing and sharing a biography of the deceased. A funeral, like a wedding, symbolizes a fundamental passage, marking the transit from life to death and allowing the codified expression of feelings. The transmission of emotions enables the expression of grief, giving it a ritualized frame of meaning (Holloway et al., 2013). This, to some extent, with the necessary involvement of the body of the mourners and their expressive capacities, stands against the backdrop of increased commercialization which define the purchase of the funeral services. The “emotional labour” (formalized, prescribed and paid) carried out by funeral professional providers has to be met by the “emotion work” (informal, negotiated, and intimate) that the mourners accomplish to participate and celebrate the death (Hochschild, 2003). As it has been stated, “social expectations as well as cultural definitions and rules (…) tell us how important our loss is; whether we have the right to grieve; and, if so, how much, how long, and in what ways can and should we do” (Brabant, 2002, p. 30). While outsourcing to funeral professionals may reduce organisational burdens, it can limit mourners’ opportunities for active participation, which is crucial for constructing a “good” funeral and maintaining continuing bonds with the deceased and among those who were also interlocked through such bond (Holloway et al., 2013). More specifically, Hochschild (2012) points out that the funeral offers the individual a specific role: the role the mourner. Mourners bring with themselves some specific “feeling rules” which allow to express grief appropriately. The funeral calls forth, facilitates and contains grief in forms that help cope with it and constitute over time the possibility of meaningful remembrance: “a funeral—write Hochschild (*Ibid.* 63 ff.)—is ideally constructed to induce spontaneous sadness and grief … In response the bereaved person generally feels that this is the right time and place to feel grief and not much else” In fact, many participants in our study recalled relatives and friends crying “hard,” “like a baby” only just at a funeral and having themselves cried “almost unexpectedly.” And indeed, many have pointed out that the funeral serves above all those left behind: “The funeral is not for the dead, it’s for the living. It is a time for consolation, for understanding, for sharing” (Int. 204). And it is a time for appropriating, though heartfelt emotions, small details and meaningful words, the services which have been previously purchased.

To be sure, precisely as a moment ceremonially dedicated to grief and its sharing, a programmed and codified pain, the funeral harbors ambivalent feelings (Szmigin and Canning, 2015). But the ambivalence of the funeral is typically taken into account, and largely managed, precisely as the social importance of collective mourning is underlined. What is more, in personalizing the funeral, mourners often find that authenticity dissolves the ambivalence of the funeral. When personalization sets in, the standard structure of the ritual becomes just that, its commercial infrastructure is often put in the background. So, for example, A., a part-time worker from Lombardy close to retirement, claims that the feelings at the funeral of her mother were as real as the involvement of all the family members in the personalization of the ceremony:

And there I never saw my brother crying like a child. And we also cry (…) All the relatives there. And we really… we started crying (…) one of my sisters went, my sister Patrizia. And she chose the coffin. As I said, my mom had chosen the dress. And… what there is to read in church and so my niece chose it instead. Because we wanted a sung funeral (…) my niece chose the songs that she wanted (Int. 33).

In this and in many other verbalizations, the mourners stress the role of intimate relations, of the memories of closeness and intimacy which allow for the details that make a funeral “special” and “really felt” while working through its highly structured character of a public ceremony.

Several studies on the United States and Great Britain (see Walter, 2020) have shown that funerals had in the past an important role especially among the newly urbanized middle class in emphasizing the social status of the family. This orientation was gradually lost in the Twentieth Century and is now less important than the desire to celebrate the life of the deceased like a particular individual by stressing intimate relationships, everyday tastes, and life accomplishments. In fact, in our interviews it is rare to find reference to funerals in terms of status. Some interviewees underlined that in the past family members might have wanted “to make a good impression,” but this is typically imagined as a memory of an older time: the interviewees often contrast the funeral that they have chosen for their loved ones, a funeral perhaps modest, simple, but heartfelt, with those few pompous and ostentatious funerals they might have attended during childhood or they have seen in films. Increasingly in advanced industrial societies funerals rather than unfolding as conspicuous consumption rituals unfold as experience and memory rituals. In other words, rather that aiming at showing the status of the family, they tend to celebrate the specific personality and in fact the singularity of the life of the deceased (Walter, 2020).

It is in this light that the personalization of the funeral is very important for Italians today. Flowers have often been evoked as a central symbolic element of the funeral, indicating life and purity. They are also often a central element of personalization, which in this case goes through the market and is included in the service offered by the funeral home. But it is above all other elements of personalization that are remembered, those that do not come through the marketplace but through the eulogies, the words, the letters read during the ceremony. *Intimate personalization* as opposed to *commercial* personalization is crucial, especially in what has otherwise been defined as a broader context of de-ritualisation of mourning (Guillard, 2017). Above all, it is the words spoken during the ceremony that many recall and refer to as underscoring the significance of the ritual and its power to establish recognition of, and remembrance for, the deceased. This practice was less widespread in the past, and some still see it as more typical of countries like the US, but it has become very consolidated in recent years, especially in the North and the Centre of Italy. It is often a matter of letters to the deceased, short writings crafted on the spur of the moment, immediately after the death for the occasion, most frequently by a family member, but also by friends and colleagues. They are read during the function and remain in the memory of the mourners for their emotional content, the ability to evoke details of the life and character of the deceased that makes him or her still alive and present, the participatory and at the same time composed way of pronouncement. As it emerges from our interviews, non-religious funerals are often more personalized in such a way than the religious (mostly Catholic) ones, in which rituals and traditional readings are chosen exclusively by the priest and follow the standard liturgy.

Eulogies require quite a bit of “emotion work” to be delivered and it is the felicity of this work that is associated to a feeling of accomplishment. In particular, accuracy and authenticity are fundamental to these memories, along with the ability to express them with an appropriately sorrowful but composed attitude (Bailey and Walter, 2016). These are words that are often described as “moving.” And they are words that as C., a Sicilian worker, says, stick in participants’ memory: “For my husband my daughter spoke. She said words that are remembered in life. Let us say that my husband was a good father” (Int. 19). Embodiment, or the effort to be composed while feeling deeply, is what stresses the authenticity of the performance. A personal recollection, read perhaps in a voice broken by emotion while standing collected in front of a large congregation, functions as a tribute and as a strongly personalized greeting that has the capacity to involve the whole congregation. In fact, it very often involves the participants with anecdotes or refers to specific characteristics of the deceased with which most may identify. This creates a memory that is relished by family members among the precious things that help maintaining the memory of the deceased and its relations to the living. Emotions need to be there and at the same time they have to be contained:

“then the reading was nice my grandfather’s, it was very dedicated, very dedicated to his daily life…to his ritual, to buying the newspaper, to going downstairs with the dog…that is, there was a lot there (…) I was able to do it because I was less emotionally involved, as much as I regretted it because he was my grandfather” (Int. 40).

Personalized addresses are remembered and perform the authenticity of the ritual as they portray a lived and peculiar, to some extent unique, image of the deceased in the little details of his life and character, well beyond the paraphernalia of the funeral.

The parish priest’s eulogies are also remembered when they are capable of being both personal and heartfelt. In some cases, of course, they are quite distant from the deceased: generic eulogies, even banal, because they do not capture his or her singularity. But when they contribute to the overall personalization of the ceremony and are the embodied expression of a heartfelt closeness, the words of the parish priest are important and remain in the memory.

It is especially the details of intimate personalization, those that come through the doings and sayings of congregants, which are remembered, alongside expressions of grief. Many people remember details of funerals where aspects of the dead person’s life come to the surface and mark the funeral through the collective involvement of the participants: so for an elderly ex-*carabiniere*^4^ we find at the funeral the *carabinieri* who “practically do an escort service,” they salute, they stop the traffic; for an amateur football coach there are all the “teams of children in uniform” to give the last salute; for a pensioner who dedicated herself to voluntary work, there are “all the volunteers” to remember the passing; for a young man killed by the Camorra there are “all the institutions.” Even a conventional funeral mass can become personal and memorable if the deceased is an active member of the parish. Thus, for example, S., a middle-aged freelance professional from Emilia, remembers the funeral of one of her pre-adolescent class mates: this was a funeral which remained memorable both because of the young age of the deceased and because the church choir to which both girls belonged accompanied the whole ritual: “she also sang in the choir of our parish, and all of us, our whole age group, were clearly all there, singing” (Int. 127).

## 6 Concluding remarks

Looking at funerals, commercialization and intimate relations has proved an excellent vantage point to consider the intermingling of intimate life and the market, and the way consumption may offer occasions for appropriation and personalization which draw on warm personal relations and heartfelt feelings. Such feelings are themselves standardized through ritual elements which are the object of negotiation and recognition. Funeral services are increasingly commercialized. The funeral industry is more and more equipped to deal with the ceremonies around death and with the body of the deceased, offering full services that allow the mourner to outsource the bulk of the practicalities of death. Outsourcing or delegating funeral services is paramount and yet it is not enough. On the contrary it is fraught with ambivalence. Commercialization, with its characteristic “coolness,” poses a predicament to the main function of funerals today, that of the personalized, emotionally thick celebration of the deceased death. In order to re-frame commercialization, and mediate what is nevertheless largely acknowledged as a necessary and useful market service, intimate personalization is fundamental. Commercial personalization, through customization and commodities provided by the industry, does not appear to be as effective as intimate personalization for the reported success of a consumer ritual such as a funeral. Families in grief are glad to outsource the funeral, and yet they feel they need to re-appropriate the ceremony in personal ways with emotionally rich details that stress its authenticity. As we have seen, intimate personalization unfolds through informal “emotion work,” which ranges from the portrayal of the peculiarity of the deceased with sombre emotional depth to the demonstration of heartfelt participation. Indeed, a numerosity is important for the perceived success of the funeral: a sizeable heartfelt participation celebrates the significance of the family and the deceased in the community.

Our research has shown the importance in contemporary Italy of a “good funeral.” What makes a funeral a “good” funeral? The elements that seem to emerge go in the direction of intimate personalization: the priest’s speech, which demonstrates direct knowledge of the deceased; readings and speeches by family members and friends; the choice of music, personalized according to the tastes of the deceased; how many people are there; their closeness as a collective and communal body and how many people presented condolences and how many were crying; elements of personalization following the ritual (toasts and speeches at the dinner, choice of objects of the deceased). Also, the presence of people close to the interviewee rather than to the deceased make a funeral a nice funeral, for example many remember with pleasure the presence of their colleagues and friends at a family funeral.

When does a funeral go wrong? When, for example, the wishes of the deceased do not seem to be respected (e.g., a religious speech in a secular funeral, or a religious funeral for an atheist deceased); or if people limit the farewell to the moment of the ceremony, when everything ends there; when the environment (especially if for non-religious funerals) does not seem adequate, or some participants or the priest appear to take too much of the scene as if they were the protagonist of the ritual.

Indeed, observing the ways funerals may be considered by mourners as successful, “good” funerals, they typically entail some form of personalization which exceeds the market boundaries and involve, directly and quite evidently, intimate life. A successful funeral is told as having been ultimately felt as “authentic.” As we know authenticity is crucial in contemporary consumer culture (Sassatelli, 2007). Today’s funerals as consumption rituals are no exception. If, in most market domains, authenticity is claimed and sustained through “otherizing” (referring to an exotic or extraordinary producer) or “traditionalizing” (referring to a long standing tradition) (Koontz, 2010), in the case of funeral service and products it is claimed and sustained through *intimate personalization*: intimate relations, with their drawing on and building of a shared memory among the participants are the main ingredients to marshal and appropriate goods and services as “authentic.” Furthermore, our findings clearly show that authenticity validate consumption as a valuable experience. In the case of funerals accounts of authenticity are part of grief management, the process of transforming the relation to the deceased (rather than removing it, as psychological accounts suggest). This in turn stresses the collective, ritual dimension as long as grief “is not what happens to the person alone, but is what happens to a relationship in which the bereaved person is implicated” (Guillard, 2017, p. 480).

Considering funerals on the backdrop of increased commercialization has helped understanding better the interplay between market and intimate relations. The funerary industry provides for most of the required services and allows for increasing measures of personalization. But in many ways, what is crucial is the “emotion work” of participants, and intimate personalization is the spice that transforms the occasion into a truly authentic celebration of the deceased. Authenticity is not in the objects and services per se but in the relations wherein consumption is embedded, and in the experiences created by participants (Miller, 1987). By exploring consumers’ own voices though empirical research, we have underlined the relevance of their experiential accounts. This has helped shedding light on the role of authenticity performance and the continuous negotiation of practices and discourses to produce meaning around it (Umbach and Humphrey, 2018). Our research has therefore granted further support to a constructivist view of authenticity. Participants in these rituals in fact, through their emotion work, perform authenticity: they certainly rely on what the market offers, on the traditional fashioning of ritual sequences, on fairly standardized commodities; and yet they navigate their experiences and strive to personalize the commercialized outsourced services they have purchased.

This explains the somewhat ambivalent relation mourners develop with the funeral industry. On the one hand they rely on commercial outsourcing and appreciate the possibility of professional customization, on the other hand, these remain, especially as if they stand alone, defined as “commercial”—and thereby in stark contrast with what is considered an authentic, and ultimately felicitous, funeral. The funeral industry may be valued because it takes quite a lot of organization weight from the shoulders of mourners and allows them to concentrate on intimate feelings, yet it is such feelings which are commanded and managed to fuel the various aspects of funeral rituals, however commercialized they may be. Feelings are thereby ritualized through various elements of the funeral ritual, as much as emotional scripts are sold by the funeral industry. Through this ambivalence, mourners perform authenticity and work on their emotions to claim authenticity in every phase of the funeral: from purchase of the services up to the actual ceremony.

Future research will be needed to explore empirically how funeral practices have evolved after the pandemic and to assess whether and how pandemic-related disruptions may have reshaped ritual forms in lasting ways. Research may thus help to understand possible new ways through which the search of authenticity might reconcile outsourcing and insourcing. As a matter of fact, the pandemic and the lockdown periods, profoundly disrupted traditional rituals of leave-taking in Italy, as elsewhere, as the need for physical distancing led to their interruption or significant alteration. This brought renewed attention to the social necessity of collective mourning and shared farewell practices: quite suddenly, precisely as they were forbidden from doing so, people re-discovered importance of being able to say goodbye to their beloved, body, and soul. In in a way, they felt appropriate and truly meaningful the embodied possibility of performing emotions. Broadly speaking, the Covid-19 experience made visible what had often been taken for granted: the importance of sharing the performance of emotions in lived consumption rituals. This was particularly salient in the case of funerals, because virtual images of disease and death were everywhere and they were typically strongly medicalized, bureaucratized and massified. People were, for a time, dispossessed of funeral rituals as embodied and shared experiences of mourning, which arguably has reinformed the relevance of embodied and shared experience. Rather than representing a definitive rupture, the post-pandemic context is one of rebounding: trends already identified in this study—such as intimate personalization—are probably bouncing back, revealing both resilience in ritual forms and the continuous relevance of insourcing on the background of increased pressure for commercialization and outsourcing. Arguably, increasingly, funeral emotion work rests on valuations of authenticity which reveals the ambivalence of the commercialization of funeral rituals.

## Data Availability

The original contributions presented in the study are included in the article/supplementary material, further inquiries can be directed to the corresponding author.

## Footnotes

1 This research was conducted before the Covid-19 pandemic (2019–2023). On the one hand, this temporal positioning is analytically relevant, as it allows us to examine funeral organization and personalization in “ordinary” conditions, before the exceptional suspension of collective rituals imposed by the lockdown in the country. On the other hand, literature suggests that its main findings remain relevant for understanding contemporary funeral practices in Italy. As a matter of fact, they refer to long-term processes related to commercialization, individualization and personalization as well as changing relationships with religious institutions, and evolving notions of identity and memory.

2 The project was carried out in years 2017–2019. The sample consisted of 405 respondents (126 men and 279 women), of whom 257 resided in Northern and Central Italy and 148 in Southern Italy and the Islands. Respondents aged under 39 were 138, those aged between 40 and 65 were 163, and those aged over 65 were 104. Overall, 214 respondents held a high school diploma, while 191 did not. A total of 244 respondents lived in municipalities with fewer than 50,000 inhabitants, whereas 161 resided in municipalities with at least 50,000 inhabitants. Moreover, since the unit of analysis was shifted from the individual to the family, it was decided to interview blood relatives belonging to different generations, such as children, parents, and grandparents. In 146 cases, two family members from different generations were interviewed, and in 32 cases three or more relatives belonging to at least three generations were interviewed. Only in 13 cases did the interviews remain isolated due to the unavailability of other family members.

3 A brotherhood (or confraternity) is a Catholic voluntary association of laypeople created for the purpose of promoting charity work. They are approved by the Church hierarchy and can enter formal concession arrangements with public Municipalities for the management of cemeterial spaces and funeral organization.

4 Carabinieri are an Italian military Corp with law enforcement duties.
